# The *In Vitro* Antimicrobial Activity of *Lavandula angustifolia* Essential Oil in Combination with Other Aroma-Therapeutic Oils

**DOI:** 10.1155/2013/852049

**Published:** 2013-05-13

**Authors:** Stephanie de Rapper, Guy Kamatou, Alvaro Viljoen, Sandy van Vuuren

**Affiliations:** ^1^Department of Pharmacy and Pharmacology, Faculty of Health Sciences, University of the Witwatersrand, Parktown, Johannesburg 2193, South Africa; ^2^Department of Pharmaceutical Sciences, Faculty of Science, Tshwane University of Technology, Pretoria 0001, South Africa

## Abstract

The antimicrobial activity of *Lavandula angustifolia* essential oil was assessed in combination with 45 other oils to establish possible interactive properties. The composition of the selected essential oils was confirmed using GC-MS with a flame ionization detector. The microdilution minimum inhibitory concentration (MIC) assay was undertaken, whereby the fractional inhibitory concentration (ΣFIC) was calculated for the oil combinations. When lavender oil was assayed in 1 : 1 ratios with other oils, synergistic (26.7%), additive (48.9%), non-interactive (23.7%), and antagonistic (0.7%) interactions were observed. When investigating different ratios of the two oils in combination, the most favourable interactions were when *L. angustifolia* was combined with *Cinnamomum zeylanicum* or with *Citrus sinensis,* against *C. albicans* and *S. aureus,* respectively. In 1 : 1 ratios, 75.6% of the essential oils investigated showed either synergistic or additive results, lending *in vitro* credibility to the use of essential oil blends in aroma-therapeutic practices. Within the field of aromatherapy, essential oils are commonly employed in mixtures for the treatment of infectious diseases; however, very little evidence exists to support the use in combination. This study lends some credence to the concomitant use of essential oils blended with lavender.

## 1. Introduction

Essential oils, which form part of naturopathic therapy, are widely known for their antimicrobial properties. They have been found to be beneficial in the fields of dermatology, gastritis, respiratory complaints, wound healing, and genital infections. Of all the essential oils used commercially, lavender (*Lavandula angustifolia*) oil appears to be one of the most popular. The earliest therapeutic use of *L. angustifolia* Mill can be traced back to Roman and Greek times [1]. The significance of which, with respect to antimicrobial applications, has been further emphasised in a number of studies [2, 3]. Within aromatherapy and wellness industries, the oil has been indicated for the treatment of a plethora of conditions, such as rhinitis, wet coughs, minor burns, and in the emergency treatment of wounds [4].

Essential oils are not only used in monotherapy but have been used in combination for many years [5]. They are used to bring about healing in a holistic manner by stimulating the mind, body, and senses, where the combination is believed to act synergistically to further enhance these effects. After examining the literature on the application of various essential oils for their use in treating microbial infections, more than 600 possible essential oil combinations were identified, of which the majority included the use of *L. angustifolia* in combination for the treatment of infections [5–11]. According to the general literature, *L. angustifolia *has been used as an antibiotic or antiseptic in combination with a number of other oils (bitter orange, caraway, cederwood, chamomile, geranium, grapefruit, lemon, marjoram, patchouli, rosemary, sage, sweet orange, and ylang-ylang) [7–9]. In spite of the numerous references to *L. angustifolia *in combination with other essential oils, supporting evidence is lacking. Only two scientific papers were found where *L. angustifolia *was studied in combination with commercially available essential oils. *Melaleuca alternifolia* (tea tree oil) in combination with *L. officinalis* was investigated against *Trichophyton rubrum* and *T. mentagrophytes* var. *interdigitale*. The data confirmed that synergistic activity was evident when these oils were placed in combination [12]. Another study conducted by Edwards-Jones et al. [13] placed *L. officinalis *in combination with *M*.* alternifolia*, *Pogostemon cablin, Pelargonium graveolens*, and Citricidal (a grapefruit seed extract, commercially available as an antibacterial agent). The antibacterial efficacy was tested using the inhibition zone method, in which direct and vapour contact of the essential oils were tested. When placed in combination with *P. graveolens* and *M. alternifolia*, *L. officinalis* demonstrated an increased inhibitory effect against *Staphylococcus aureus. *Antagonism was noted for the combination of *L. officinalis* and *M. alternifolia* against methicillin-resistant *S*.* aureus *(MRSA). While these two studies provide confirmation of improved efficacy in some cases when the oils are used in combination, the overall interactive potential with other essential oils has not been fully explored. In aromatherapy practices, essential oils are rarely used individually but rather applied as blends to achieve a superior therapeutic effect. With this in mind, the aim of this study was to evaluate the interactive* in vitro *antimicrobial properties of *L. angustifolia *and a selection of essential oils commonly used in therapeutic combinations.

## 2. Materials and Methods

### 2.1. Essential Oil Selection and Chemical Composition Analysis

Forty-five essential oil samples were obtained as a gift from commercial fragrance and flavour suppliers. The composition of the selected essential oils was confirmed using gas chromatography coupled to a mass spectrometer and flame ionization detector (GC-MS-FID). The GC-MS-FID (Agilent 6890 N GC system and 5973 MS) was equipped with a HP-Innowax polyethylene glycol column (60 m × 250 *μ*m i.d. × 0.25 *μ*m film thickness). A volume of 1 *μ*L of the essential oil was injected (using a split ratio of 200 : 1) with an autosampler at 24.79 psi and an inlet temperature of 250°C. The GC oven temperature was set at 60°C for 10 min, then 220°C at a rate of 4°C/min for 10 min and followed by a temperature of 240°C at a rate of 1°C/min. Helium was used as a carrier gas at a constant flow of 1.2 mL/min. Spectra was obtained on electron impact at 70 eV, scanning from 35 to 550 *m*/*z*. The percentage composition of the individual components was quantified by integration measurements, using flame ionization detection (FID, 250°C). Component identifications were made by comparing mass spectra from the total ion chromatogram, and retention indices using NIST and Mass Finder GC-MS libraries and major compounds given in Table 1 [14].

### 2.2. Antimicrobial Assays

Seven laboratory cultured bacterial strains, three laboratory cultured fungal strains, and four clinical bacterial strains were selected for the study (Table 2). This was undertaken to provide a broad-spectrum profile of the antimicrobial activity for* L. angustifolia. *In order to evaluate combined efficacies, three microorganisms were selected (*Staphylococcus aureus,* ATCC 6538; *Pseudomonas aeruginosa, *ATCC 27858 and *Candida albicans,* ATCC 10231), whereby the oils were evaluated for antimicrobial activity using the microdilution minimum inhibitory concentration (MIC) assay [15]. Essential oils are typically used to treat topical and respiratory infections, and these microorganisms were selected on the basis of this pathogenesis. Furthermore, selection was based on the criteria to include a Gram-positive, a Gram-negative, and a yeast strain. The Clinical and Laboratory Standards Institute guidelines (CLSI) [16] were used to ensure that accurate microbiological assay and transfer techniques were followed. Stock cultures were retained at −20°C, subcultured onto Tryptone Soya (TSA) agar, and incubated at optimum temperatures (37°C for 24 h for bacteria, and 37°C for 48 h for the yeast). Isolated pure colonies were selected and transferred onto TSA and thereafter kept viable by subculturing weekly for stock culture maintenance.

Essential oils were diluted to a concentration of 32 mg/mL using acetone as the diluent. The microtitre plates were prepared by adding 100 *μ*L of sterile, distilled water into each of the wells. Thereafter, the oils were added at a volume of 100 *μ*L (when tested individually) and 50 : 50 *μ*L (when tested in combination). The essential oils were serially diluted to yield concentrations of 8, 4, 2, 1, 0.5, 0.25, 0.125, and 0.0625 mg/mL. The microorganisms for testing were diluted using sterile TSB at a 1 : 100 dilution in order to achieve an approximate concentration of 1 × 10^6^ colony forming units (CFU)/mL. Cultures were added to all the wells of their respective microtitre plates, at a volume of 100 *μ*L. The microtitre plates were then sealed with a sterile adhesive sealing film, to prevent any essential oil loss due to their inherent volatility. The microtitre plates were incubated under optimal conditions (37°C for 24 h for bacteria and 37°C for 48 h for yeasts). After incubation, 0.4 mg/mL of *p-*iodonitrotetrazolium violet solution (INT) was added to each well (40 *μ*L). Viable micro-organisms interact with INT to create a colour change from clear to a red-purple colour. Thus the lowest dilution with no colour change was considered as the MIC for that oil [17].

For the 1 : 1 combinations, the fractional inhibitory concentration index (ΣFIC) was used to determine the interaction of the oils. The ΣFIC was calculated by dividing the MIC value of the combined essential oils with the MIC value of each essential oil placed in the combination. The ΣFIC was then calculated by adding these two values. The ΣFIC for each combination was interpreted as antagonistic where a ΣFIC value of greater than 4.00 is observed. Indifference was noted for ΣFIC values greater than 1.00 but less than or equal to 4.00. Additive properties for ΣFIC values more than 0.50 but less than or equal to 1.00, with synergistic properties noted for ΣFIC values less than or equal to 0.50 [18].

Isobolograms were constructed to determine what antimicrobial interactions could be apparent if variable concentrations of selection of oils (*Daucus carota*, *Juniperus virginiana*, *Cinnamomum zeylanicum*, and *Citrus sinensis*) were combined with *L. angustifolia*. Selection was based on promising synergistic interactions observed in the 1 : 1 ΣFIC analysis. Nine ratios, that is, 9 : 1; 8 : 2; 7 : 3; 6 : 4; 5 : 5; 4 : 6; 3 : 7; 2 : 8; and 1 : 9 of the oils were mixed and thereafter the MIC values were determined for these combinations, as well as for the essential oils independently. Isobolograms were plotted using GraphPad Prism, version five software to present the mean MIC values of the combinations as ratios [19]. The isobolograms were interpreted by examining the data points for each ratio in relation to the MIC values for the oils independently. All points between the 1.0 : 1.0 line and 4.0 : 4.0 line were classified as non-interactive. Points between the 0.5 : 0.5 and 1.0 : 1.0 line were interpreted as additive and points below or on the 0.5 : 0.5 line on the isobologram were interpreted as synergistic. Antagonism was identified as data points above the 4.0 : 4.0 line [18].

Positive and negative controls were included in all assays, with 0.01 mg/mL ciprofloxacin used as a positive control for bacteria, and 0.10 mg/mL amphotericin B for the yeast. The negative control was a water/acetone solution at a concentration of 32 mg/mL, to determine any antimicrobial activity of the diluent. Media and culture controls, such as Tryptone Soya broth (TSB), were included to confirm sterility and viability, respectively. Assays were done in triplicate and further repetitions conducted where necessary.

## 3. Results

### 3.1. Chemical Composition

The chemical composition of the essential oils were analysed in order to confirm the specific chemotypes (Table 1). Although a full chemical profile was established for each essential oil, only the major constituents have been noted for the sake of brevity. The major chemical compounds identified for the essential oils are congruent with previously published profiles. 

### 3.2. Antimicrobial Analysis

The antimicrobial efficacy for *L. angustifolia* essential oil was investigated against 14 pathogens whereby MIC values of 2.00 mg/mL were predominantly observed against the tested pathogens. A few exceptions (*Klebsiella pneumoniae *with an MIC of 1.50 mg/mL, *Candida tropicalis *with an MIC of 0.75 mg/mL, and *C. albicans *with an MIC of 3.00 mg/mL) were noted (Table 2). When examining the entire panel of oils tested, *Santalum album* showed the greatest antimicrobial effect, with the lowest MIC values for *S. aureus* (MIC value of 0.25 mg/mL) and *P. aeruginosa *(MIC value of 0.50 mg/mL).

**Figure 1:**
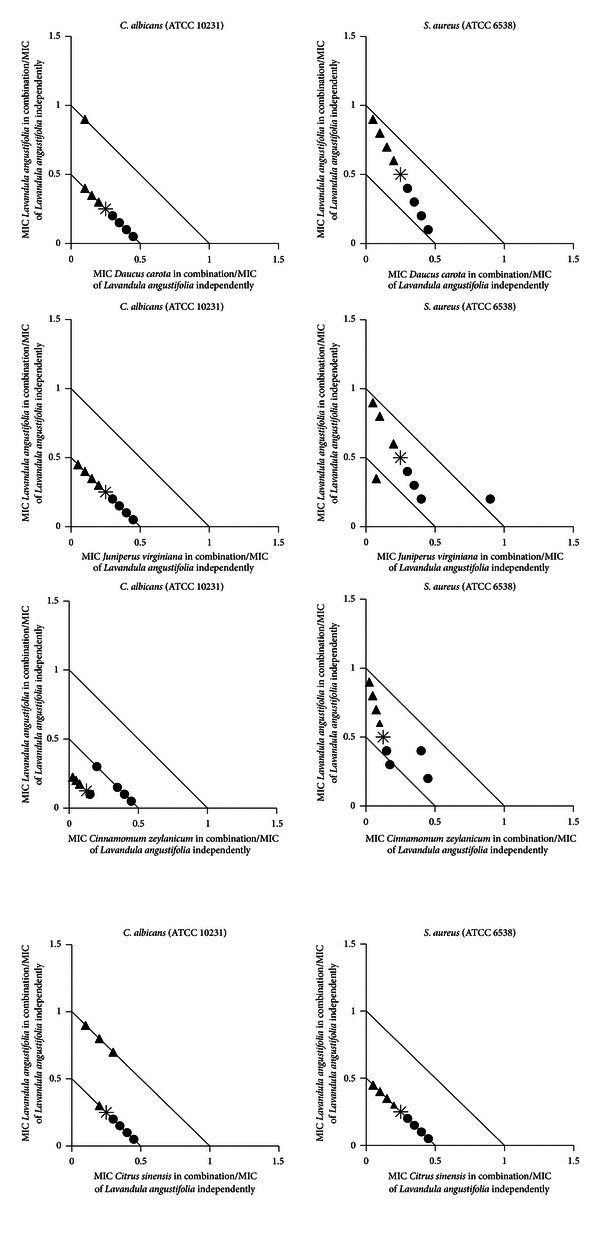
Isobolograms constructed for the synergistic essential oil combinations against the pathogens *C. albicans* and *S. aureus.* ▲ indicates *L. angustifolia *essential oil in majority concentration, ● indicates other essential oils (*D. carota* or *J. virginiana* or *C. zeylanicum* or *C. sinensis*) in majority concentration, and ∗ indicates equal ratios of essential oil in combination.

When the 45 essential oil samples were placed in combination with *L. angustifolia* in equal ratios, 75.6% of the combinations exhibited either synergistic or additive antimicrobial activity (Table 3). The most noteworthy synergistic interactions were evident for *C. albicans, *particularly the combination of *L. angustifolia *with *Cupressus sempervirens* (ΣFIC value of 0.15) and *L. angustifolia *with *Litsea cubeba* (ΣFIC value of 0.19). For all combinations studied, antagonism was only noted once, where *Cymbopogon citratus *was investigated in combination with *L. angustifolia* (ΣFIC value of 6.67). Some 1 : 1 combinations (*L. angustifolia *with either *D. carota *or* J. virginiana *or* C. zeylanicum *or *C. sinensis*) demonstrated synergistic interactions against both *C. albicans *and *S. aureus. *These combinations were analysed further against these microorganisms to determine if varied ratios of the two essential oils in the combination would yield varied interactions (Figure 1). The combination where *L. angustifolia *was combined with *C. zeylanicum *in various ratios against *C. albicans *displayed the greatest synergistic effect of all oils studied.

## 4. Discussion

Although a number of *in vitro *studies have been conducted on the antimicrobial activity of *L. angustifolia *essential oil against a wide variety of microorganisms, many studies have used disc diffusion assays to quantify antimicrobial activity, which later has been found to be largely inappropriate [20, 21]. The MIC method used for antimicrobial analysis of the essential oils is considered to be the preferred method, and as such, is the only one considered for comparative purposes here [22].

*L. angustifolia *has been extensively studied for antimicrobial effects against a variety of test microorganisms [23–33]. These previous studies have demonstrated the efficacy of this essential oil as an antimicrobial agent as well as augment the findings of this study. The essential oil from *S. album* has demonstrated notable activity in past studies. Superior antimicrobial activity (MIC value of 0.06% v/v) of *S. album *was observed when tested with a selection of 24 essential oils [25]. A later study by the same authors demonstrated activity for an Australian based* S. album* against *C. albicans *(MIC value of 0.06% v/v), *P. aeruginosa* (MIC value of >2.00 mg/mL), and *S. aureus* (MIC value of 0.12 mg/mL) [26]. The data from these studies are congruent with that reported here and demonstrate the antimicrobial potential of *S. album.*

The combination of *L. angustifolia *with *D. carota *against *C. albicans *in various ratios displayed a predominantly synergistic effect for eight of the ratios studied. For *S. aureus*, the tested ratios indicated mostly additive interactions. Where a higher concentration of *D. carota* essential oil was present in the oil mixture, a more favourable antimicrobial effect was noted. *Lavandula angustifolia *has been associated with the treatment of fungal infections and is also used in the treatment of Staphylococcal-related infections, such as boils and abscesses [6, 7]. No information could be obtained to support the use of *D. carota *for Staphylococcal and Candidal related infections, yet when the two essential oils are combined, the ratios where *D. carota *is in higher concentrations demonstrated a more favourable effect. This suggests that the presence of *D. carota *plays a role in the additive and synergistic findings observed against these microorganisms tested. 

The combination of *L. angustifolia *and *J. virginiana *against *C. albicans *in various ratios indicated a synergistic effect for all nine of the ratios analysed. Against *S. aureus*, the ratios displayed a predominantly additive effect. *Juniperus virginiana *in combination with *L. angustifolia *essential oil has been implicated for the treatment of bacterial respiratory infections [5], while the combination was also noted for its use in the treatment of Candidal-type infections such as thrush [7]. Where the two essential oils are combined, regardless of ratio, the essential oils display synergistic effects for *C. albicans*. 

The combination where *L. angustifolia *was combined with *C. zeylanicum *in various ratios against *C. albicans *displayed the greatest synergistic effect of all oils studied. It was also interesting to note that for these combinations, the higher the concentration of *L. angustifolia, *the more favourable (additive or synergistic) the interaction.Against *S. aureus*, the converse was true. Most of the ratios indicated an additive effect with one ratio (*L. angustifolia*:* C. zeylanicum*, 3 : 7) indicating synergy. The combination of *C. zeylanicum *and *L. angustifolia* has been associated with the treatment of topical infections and as a general antimicrobial agent [8]. This combination has demonstrated a greater antifungal effect when placed in various ratios as five of the nine ratios were synergistic against *C. albicans*. The ratios where *L. angustifolia *is in higher concentrations demonstrated a more favourable effect and this suggests that the presence of *L. angustifolia* plays a greater role in the synergy observed. 

When *L. angustifolia *was combined with *C. sinensis *in various ratios, a predominantly synergistic effect was recorded for both microorganisms tested. The use of *C. sinensis *essential oil in combination with *L. angustifolia *for the treatment of respiratory infections has been documented [8]. Even though *C. sinensis *demonstrated poor activity when tested singularly [34, 35], when combined with *L. angustifolia, *efficacy was enhanced. 

## 5. Conclusion

In summary, the analysis of the combination of *L. angustifolia *with a selection of other aroma-therapeutic essential oils has largely demonstrated noteworthy activity against the pathogens tested. The ΣFIC analysis indicated that these oils have favourable antimicrobial interactions when placed in combination, that is, 26.7% synergistic and 48.9% additive effects for selected oils. Only one combination (*C. citratus *in combination with *L. angustifolia* with a ΣFIC value of 6.67) demonstrated antagonistic effects. When placed in various ratios the combination of *L. angustifolia *and *C. sinensis *essential oil demonstrated the best antimicrobial effect with synergy identified for all ratios against the microorganisms tested. Essential oils blends have been used for many years for their curative properties and this study lends credibility to the frequent use of combining oils to achieve a superior therapeutic outcome. 

## Figures and Tables

**Table 1:** The chemical composition (major compounds only) of the essential oils under investigation.

Essential oil	Major constituent	% Abundance
*Abies balsamea *	**β*-*pinene	31.0
	bornyl acetate	14.9
	**δ**-3-carene	14.2

*Andropogon muricatus *	*β*-vetirenene	7.2
	zizanol	12.6
	*λ*-vetivenene	4.5
	isoeremophilene	3.2
	*β*-vetispirene	3.2
	vetivenic acid	3.7

*Angelica archangelica* root	**α**-phellandrene	18.5
	**α**-pinene	13.7
	**β**-phellandrene	12.6
	**δ**-3-carene	12.1

*Angelica archangelica* seed	**β**-phellandrene	59.2

*Anthemis nobilis *	2-methylbutyl-2-methyl propanoic acid	31.5
	limonene	18.3
	3-methylpentyl-2-butenoic acid	16.7
	isobutyl isobutyrate	10.0

*Artemisia dracunculus *	estragole	82.6

*Canarium luzonicum *	limonene	41.9
	elemol	21.6
	**α**-phellandrene	11.4

*Cananga odorata *heads	benzyl acetate	31.9
	linalool	27.0
	methyl benzoate	10.4

*Cananga odorata *	bicyclosesquiphellandrene	19.5
	**β*-*farnesene	13.9

*Carum carvi *	limonene	27.6
	carvone	67.5

*Cinnamomum zeylanicum *	eugenol	80.0

*Citrus aurantium *	linalyl acetate	54.9
	linalool	21.1

*Citrus grandis *	limonene	74.8

*Citrus sinensis *	limonene	93.2

*Commiphora myrrha *	furanoeusdema-1,3-diene	57.7
	lindestrene	16.3

*Cupressus sempervirens *	**α**-pinene	41.2
	**δ**-3-carene	23.7

*Cymbopogon citratus *	geranial	44.8

*Cymbopogon nardus *	citronellal	38.3
	geraniol	20.7
	citronellol	18.8

*Daucus carota *	carotol	44.4
	**β**-caryophyllene	5.7
	**β**-bisabolene	5.3

*Eucalyptus globulus *	1,8-cineole	58.0
	**α*-*terpineol	13.2

*Foeniculum dulce *	*E*-anethole	79.1

*Hyssopus officinalis *	isopinocamphone	48.7
	pinocamphone	15.5

*Juniperus virginiana *	thujopsene	29.8
	cedrol	14.9
	**α**-cedrene	12.4

*Juniperus virginiana *berries	**α**-pinene	20.5
	myrcene	13.7
	bicyclosesquiphellandrene	10.7

*Laurus nobilis *	eugenol	57.2
	myrcene	14.3
	chavicol	12.7

*Lavandula angustifolia *	linalyl acetate	36.7
	linalool	31.4
	terpinen-4-ol	14.9

*Litsea cubeba *	geranial	45.6
	nerol	31.2

*Matricaria chamomilla *	bisabolene oxide A	46.9
	**β*-*farnesene	19.2

*Melaleuca alternifolia *	terpinen-4-ol	49.3
	*γ-*terpinene	16.9

*Melaleuca viridiflora *	1,8-cineole	45.9
	**α*-*terpinene	21.0

*Mentha piperita *	menthol	47.5
	menthone	18.6

*Myrtus communis *	myrtenyl acetate	28.2
	1,8-cineole	25.6
	**α**-pinene	12.5

*Ocimum basilicum *	linalool	54.1

*Origanum majorana *	1,8-cineole	46.0
	linalool	26.1

*Pelargonium odoratissimum *	citronellol	34.2
	geraniol	15.7

*Pinus sylvestris *	bornyl acetate	42.3
	camphene	11.8
	**α**-pinene	11.0

*Piper nigrum *	**β**-caryophyllene	33.8
	limonene	16.4

*Pogostemon patchouli *	patchouli alcohol	37.3
	**α*-*bulnesene	14.6
	**α*-*guaiene	12.5

*Rosmarinus officinalis *	1,8-cineole	48.0

*Salvia sclarea *	linalyl acetate	72.9
	linalool	11.9

*Santalum album *	**α*-*santalol	32.1

*Styrax benzoin *	cinnamyl alcohol	44.8
	benzene propanol	21.7

*Syzygium aromaticum *	eugenol	82.2
	eugenol acetate	13.2

*Tagetes patula *	(*E*)-**β**-ocimene	41.3
	*E*-tagetone	11.2
	verbenone	10.9

*Thymus vulgaris *	*p*-cymene	39.9
	thymol	20.7
	*γ-*terpinene	8.3

**Table 2:** The antimicrobial effects of *L. angustifolia *essential oil against 14 test pathogens.

Microorganism	Reference strain no.	MIC*
Methicillin-resistant *Staphylococcus aureus *(MRSA)	ATCC 43300	2.00
Methicillin-resistant *Staphylococcus aureus *	Clinical strain	2.00
Methicillin-gentamicin resistant *Staphylococcus aureus *(MGRSA)	ATCC 33592	2.00
*Staphylococcus aureus *	ATCC 6538	2.00
*Staphylococcus aureus *	Clinical strain	2.00
*Staphylococcus epidermidis *	ATCC 2223	2.00
*Staphylococcus epidermidis *	Clinical strain	2.00
Vancomycin-resistant* Enterococcus faecalis *	ATCC 51299	2.00
*Enterococcus faecalis *	Clinical strain	2.00
*Klebsiella pneumoniae *	ATCC 13883	1.50
*Pseudomonas aeruginosa *	ATCC 27853	2.00
*Cryptococcus neoformans *	ATCC 11093	2.00
*Candida tropicalis *	ATCC 201380	0.75
*Candida albicans *	ATCC 10231	3.00
Ciprofloxacin	Positive control	0.20 × 10^−3 ^to 0.60 × 10^−3^
Amphotericin B	Positive control	0.60 × 10^−3^

*MIC given in mg/mL.

**Table 3:** The antimicrobial effects of the essential oils studied individually and in combination with *L. angustifolia *essential oil.

Essential oil	Oils examined individually			1 : 1 Combinations					
	*C. albicans *	*S. aureus *	*P. aeruginosa *	*C. albicans *		*S. aureus *		*P. aeruginosa *	
	(ATCC 10231)	(ATCC 6538)	(ATCC 27858)	(ATCC 10231)		(ATCC 6538)		(ATCC 27858)	
	MIC	MIC	MIC	MIC	ΣFIC	MIC	ΣFIC	MIC	ΣFIC
*Abies balsamea *	2.00	3.00	2.00	1.50	0.63	6.00	2.50	1.00	0.52
*Andropogon muricatus *	1.75	0.75	1.50	1.00	**0.45**	1.00	0.92	2.00	1.02
*Angelica archangelica *(seed)	2.00	2.00	2.00	2.00	0.83	4.00	2.00	1.00	0.75
*Angelica archangelica *(root)	2.00	1.75	2.00	1.00	**0.42**	2.00	1.07	1.00	0.67
*Anthemis nobilis *	3.00	16.00	2.00	1.00	**0.33**	3.00	0.84	1.00	0.54
*Artemisia dracunculus *	2.00	3.00	2.00	1.00	**0.42**	4.00	1.67	1.00	0.51
*Canarium luzonicum *	3.00	3.00	2.00	0.75	**0.25**	8.00	3.33	1.00	0.53
*Cananga odorata *(heads)	2.00	4.00	1.50	2.00	0.83	3.00	1.13	2.00	1.02
*Cananga odorata *	2.00	2.00	3.00	3.00	1.25	3.00	1.50	2.00	1.02
*Carum carvi *	2.00	2.00	2.00	1.00	**0.42**	2.00	1.00	1.00	0.56
*Cinnamomum zeylanicum *	2.00	2.00	1.50	1.00	**0.40**	1.00	**0.50**	1.00	0.53
*Citrus aurantium *	2.00	4.00	2.00	1.00	**0.42**	3.00	1.13	1.00	0.51
*Citrus grandis *	2.00	3.00	1.50	1.00	**0.42**	4.00	1.67	1.00	0.52
*Citrus medica limonum *	2.00	3.00	2.00	1.00	**0.42**	6.00	2.50	1.00	0.52
*Citrus sinensis *	2.00	4.00	2.00	1.00	**0.42**	1.00	**0.38**	1.00	0.51
*Commiphora myrrha *	4.00	2.00	4.00	1.00	**0.29**	2.00	1.00	2.00	1.03
*Cupressus sempervirens *	4.00	12.00	2.00	0.50	**0.15**	2.00	0.58	1.00	0.53
*Cymbopogon citratus *	2.00	1.67	1.50	0.50	6.67	1.00	0.55	1.00	0.52
*Cymbopogon nardus *	0.75	4.00	1.50	0.75	**0.42**	2.00	0.75	1.00	0.53
*Daucus carota *	3.00	2.00	3.00	1.50	**0.50**	1.00	**0.50**	1.00	0.56
*Eucalyptus globulus *	1.50	4.00	3.00	0.75	**0.38**	4.00	1.50	1.00	0.53
*Foeniculum dulce *	2.00	2.00	3.00	1.00	**0.45**	4.00	2.00	1.00	0.52
*Hyssopus officinalis *	1.00	3.00	2.00	0.50	**0.33**	4.00	1.67	1.00	0.52
*Juniperus virginiana *	1.50	2.00	2.00	1.00	**0.50**	1.00	**0.50**	1.00	0.55
*Juniperus virginiana* (berries)	2.00	3.00	2.00	0.50	**0.21**	3.00	1.25	1.00	0.52
*Laurus nobilis *	0.75	0.83	2.67	1.00	0.83	2.00	1.70	1.00	0.60
*Litsea cubeba *	6.00	1.50	1.50	0.75	**0.19**	2.00	1.17	1.00	0.52
*Matricaria chamomilla *	0.50	1.50	4.00	1.00	1.17	2.00	1.17	1.00	0.54
*Melaleuca viridiflora *	1.75	2.00	2.00	2.00	0.90	4.00	2.00	1.00	0.51
*Melaleuca alternifolia *	1.50	8.00	2.00	1.00	**0.50**	2.00	0.63	1.00	0.51
*Mentha piperita *	2.00	4.00	2.00	1.50	0.63	2.00	0.75	1.00	0.51
*Myrtus communis *	1.50	2.00	2.00	1.00	**0.50**	2.00	4.00	1.00	0.51
*Ocimum basilicum *	1.00	1.50	2.67	1.00	0.67	1.00	0.58	1.00	0.63
*Origanum majorana *	2.00	2.00	2.00	1.00	**0.42**	2.00	4.00	1.00	0.52
*Pelargonium odoratissimum *	0.75	1.50	2.00	1.25	1.04	2.00	1.17	1.00	0.52
*Pinus sylvestris *	1.50	4.00	2.00	1.00	**0.50**	2.00	0.75	2.00	1.00
*Piper nigrum *	2.00	2.00	2.00	1.00	**0.42**	2.00	1.00	1.00	0.57
*Pogostemon patchouli *	1.50	1.50	2.00	1.00	**0.50**	2.00	1.17	1.00	0.51
*Rosmarinus officinalis *	2.00	4.00	2.00	1.00	**0.42**	2.00	0.75	1.00	0.51
*Salvia sclarea *	0.88	2.00	3.50	1.00	0.73	2.00	1.00	1.00	0.51
*Santalum album *	2.00	0.25	0.50	1.00	**0.42**	1.00	2.25	1.00	0.51
*Styrax benzoin *	2.00	2.00	3.00	1.00	**0.42**	2.00	1.00	1.00	0.58
*Syzygium aromaticum *	0.50	1.50	1.50	0.50	0.58	2.00	1.17	1.00	0.53
*Tagetes patula *	2.00	4.00	1.50	1.00	**0.42**	2.00	0.75	1.00	0.51
*Thymus vulgaris *	1.00	3.33	2.00	1.00	0.67	1.00	**0.40**	1.00	0.51

Ciprofloxacin control	NR*	0.45 × 10^−3^	0.45 × 10^−3^	NR*		0.45 × 10^−3^		0.45 × 10^−3^	
Amphotericin B control	0.60 × 10^−3^	NR*	NR*	0.60 × 10^−3^		NR*		NR*	

*NR indicates not relevant; bold indicates synergy; MIC given in mg/mL.
